# Comparative Analysis of Glycogene Expression in Different Mouse Tissues Using RNA-Seq Data

**DOI:** 10.1155/2014/837365

**Published:** 2014-07-09

**Authors:** Ahmad Firoz, Adeel Malik, Sanjay Kumar Singh, Vivekanand Jha, Amjad Ali

**Affiliations:** ^1^School of Chemistry and Biochemistry, Thapar University, Patiala, Punjab 147004, India; ^2^Biomedical Informatics Center of ICMR, Post Graduate Institute of Medical Education and Research (PGIMER), Chandigarh 160012, India; ^3^School of Biotechnology, Yeungnam University, 280 Daehak-ro, Gyeongsan, Gyeongbuk 712-749, Republic of Korea; ^4^Department of Biotechnology, Thapar University, Patiala, Punjab 147004, India; ^5^Department of Nephrology, Post Graduate Institute of Medical Education and Research (PGIMER), Chandigarh 160012, India

## Abstract

Glycogenes regulate a wide array of biological processes in the development of organisms as well as different diseases such as cancer, primary open-angle glaucoma, and renal dysfunction. The objective of this study was to explore the role of differentially expressed glycogenes (DEGGs) in three major tissues such as brain, muscle, and liver using mouse RNA-seq data, and we identified 579, 501, and 442 DEGGs for brain versus liver (BvL579), brain versus muscle (BvM501), and liver versus muscle (LvM442) groups. DAVID functional analysis suggested inflammatory response, glycosaminoglycan metabolic process, and protein maturation as the enriched biological processes in BvL579, BvM501, and LvM442, respectively. These DEGGs were then used to construct three interaction networks by using GeneMANIA, from which we detected potential hub genes such as *PEMT* and *HPXN* (BvL579), *IGF2* and *NID2* (BvM501), and *STAT6* and *FLT1* (LvM442), having the highest degree. Additionally, our community analysis results suggest that the significance of immune system related processes in liver, glycosphingolipid metabolic processes in the development of brain, and the processes such as cell proliferation, adhesion, and growth are important for muscle development. Further studies are required to confirm the role of predicted hub genes as well as the significance of biological processes.

## 1. Introduction

Cell function within an organism has huge variation due to gene expression pattern although all cells in an individual mammal have almost identical DNA [1]. It is important to know how cells and tissues differ in gene expression which is regulated during developmental changes in different tissues, consequently affecting their biological functions. The pattern of glycogene expression is one of important factors that provide clues about many biological functions, developmental changes, and diseases in human, mouse, and tissues of other organisms [2–4]. Glycogene is a gene that is responsible for the glycosylation of proteins, lipids, and proteoglycans and includes genes associated with the synthesis of glycans such as glycosyltransferase, sugar-nucleotide transporters, and sulfotransferases [5, 6]. Similarly, the term glycogenome involves all genes that play a role in glycosylation and represents more than 600 genes in the mouse genome [7]. Glycosylation is one of the most common posttranslational modification reactions [8]. The glycogene expression is relatively weak as compared to other molecules; however, different glycogenes are upregulated by specific tissues depending on the local conditions [5].

In order to understand the pathology of various diseases, recently, many studies have targeted and explored the role of glycogenes expression in various diseases [9–12]. Alteration in the glycogene expression is also associated with drug resistance of various types of cancers including pancreatic cancer epithelial-mesenchymal transition [13], breast cancer [14], and hepatocarcinoma [15]. The change in the glycogene expression is also associated with conjunctival epithelium [2] and primary open-angle glaucoma (POAG) [16]. Moreover, the defects in the normal expression of glycogenes in tonsillar B lymphocytes are also correlated with the proteinuria and renal dysfunction in IgA nephropathy [17, 18].

Several high-throughput microarray studies have been applied to investigate the functional roles of various glycogenes [11, 19, 20]. However, to the best of our knowledge, no study till date has utilized RNA-Seq data in the existing databases to specifically explore the role of glycogenes. Previously, we identified the role of glycogenes in skeletal muscle development in MYOG_kd_ cells by using RNA-Seq data. Therefore, in this work, we intend to investigate the role of glycogenes in various biological processes that are involved in the development of brain, muscle, and liver tissues using the precomputed expression values from mouse RNA-seq data. Functional and pathway analysis of differentially expressed glycogenes (DEGGs) between BvL579, BvM501, and LvM442 were enriched with gene ontology (GO) terms and Kyoto Encyclopedia of Genes and Genomes (KEGG) pathways, respectively. Additionally, the interaction networks of DEGGs were constructed to identify the hub genes as well as to detect the functional modules in these networks by using community analysis. We expect that the findings of our study may shed new lights on the roles of glycogenes in the development of these tissues and their pathogenesis.

## 2. Materials and Methods

### 2.1. Datasets

The precomputed expression values with no spike for the brain, liver, and muscles C57BL6 mouse tissues were downloaded from WoldLab (http://woldlab.caltech.edu/~alim/RNAseq/) for our computational analysis. These precomputed expression values represent the RNA-seq analysis initially performed by Mortazavi et al., using Illumina Genome Analyzer and expression values computed by using ERANGE package [21]. To remove low expressed genes the dataset was filtered by removing genes with RPKM value <1 in all the three tissues.

### 2.2. Fold Change Analysis

Fold change is often used in analysis of gene expression data of RNAseq experiments, for measuring change in the expression level of a gene and comparing the expression of genes between two sets of arrays, for example, case and control sets. Fold change analysis of genes commonly expressed between two different samples was done to compare the gene expression in glycogenes [22, 23]. Fold change was calculated as the ratio of brain versus liver, brain versus muscle, and liver versus muscle groups. Additionally, to further investigate the role of glycogenes in different tissues, the differentially expressed genes were manually verified in the UniProt database [24] to check whether they represent a glycogene or not. A gene was considered as a glycogene if it was annotated as a “glycoprotein” in the “Keywords” section of the UniProt database.

### 2.3. Functional Analysis

DAVID (http://david.abcc.ncifcrf.gov/home.jsp) functional annotation cluster analysis was performed on the list of differentially expressed glycogenes (DEGGs) with a fold change of ≥2. Only those terms that reported a *P* value of ≤0.05 and count number ≥5 genes were selected for analysis. The gene ontology (GO) term biological process (BP) in DAVID was used to categorize enriched biological themes in the list of DEGGs. Pathway mapping was performed using the KEGG Automatic Annotation Server (KAAS) (http://www.genome.jp/kegg/kaas/) [25]. The amino acid sequences of these DEGGs were uploaded to the KAAS web server as an input using single-directional best hit (SBH) method to assign orthologs. KAAS offers functional annotation of genes in a genome via a BLAST similarity searches against a manually curated set of ortholog groups in the KEGG GENES database. KAAS assigned a KEGG orthology (KO) number to genes in the data sets, which were mapped to one of KEGG's reference pathways.

### 2.4. Network Analysis

The functional interactions between DEGGs were analyzed by GeneMANIA webserver [26]. The GO term biological process was used to create the interaction network between the DEGGs and 50 additional genes by using mouse as a source species. The relationship between the genes in the network includes coexpression, physical and genetic interactions, pathways, colocalization, protein domain similarity, and predicted interactions. The network was filtered by removing all the interactions where weights <0.1.

### 2.5. Identification of Hub Genes

Generally, the biological networks exhibit the scale-free property [27] where only a few nodes in the network have many connections that represent hubs in the network. Hub genes were identified by calculating the node degree distribution [28] by using the NetworkAnalyzer plugin of Cytoscape. A glycogene with the highest degree distribution was considered as a hub in the current network.

### 2.6. Community Analysis

The functional modules within the network were further detected by using the greedy community-structure detection algorithm, implemented in the Cytoscape plugin GLay (http://brainarray.mbni.med.umich.edu/sugang/glay/) [29]. GLay offers Cytoscape users a diverse group of community structure algorithms and graph layout functions for network clustering and structured visualization [29]. To identify the overrepresented biological functions within each cluster, the detected clusters were subjected to functional enrichment analyses by DAVID functional analysis tool. For enrichment analysis, only those communities were analyzed which have at least 10 nodes.

## 3. Results

### 3.1. Identification of Differentially Expressed Glycogenes (DEGGs)

**Figure 1:**
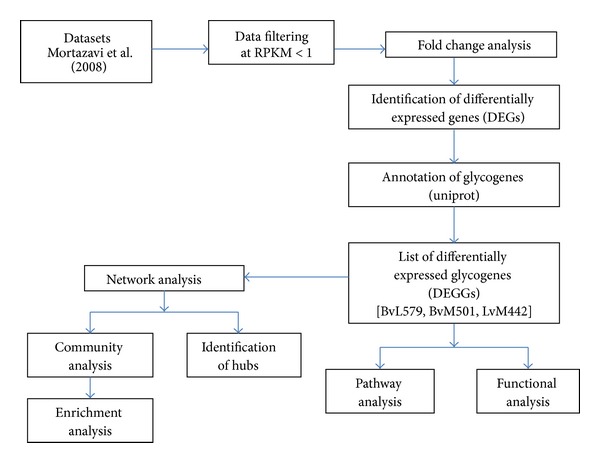
Flowchart depicting the overall methodology adopted in this study.

We downloaded the precomputed RPKM values for mouse transcriptome that was mapped and quantified previously by RNA-Seq analysis [21]. This RNA-Seq data consists of RNA from adult mouse brain, liver, and skeletal muscle tissues and represents 33598 genes in each tissue sample. The data was further filtered by excluding the genes with RPKM values <1 from the analysis. As a result, 15,237, 11,920, and 12,202 genes were identified from brain, liver, and muscle samples, respectively (Table 1(a)). Then three groups (brain versus liver, brain versus muscle, and liver versus muscle) were created and the number of genes that were common in each group were identified and summarized in Table 1(b). From the table, it can be observed that the number of common genes between brain versus liver is 10653, brain versus muscle is 10820, and liver versus muscle is 9833, respectively. These shared genes in each group were then used to calculate the fold change, which was defined as the ratio of RPKM values of brain versus liver, brain versus muscle, and liver versus muscle groups. In this study, the total fold change of ≥2 was considered to classify the differentially expressed genes (DEGs). Based on this definition, there are 5228 DEGs between brain versus liver, 5283 between brain versus muscle, and 3840 between liver versus muscle. The list of DEGs in each group was further filtered by retaining only those genes that were annotated as a “glycoprotein” in the UniProt database. Therefore, 579, 501, and 442 differentially expressed glycogenes (DEGGs) for brain versus liver (BvL579), brain versus muscle (BvM501), and liver versus muscle (LvM442) groups were selected for the final analysis (Table 1(b)). The complete workflow of the analysis is shown in Figure 1.

### 3.2. Functional Annotation and Pathway Analysis

To classify biological processes that are enriched in the BvL579, BvM5, Annotation Cluster (FAC) tool available in the Database for Annotation, Visualization, and Integrated Discovery (DAVID) (http://david.abcc.ncifcrf.gov/home.jsp). The GO term “Biological Process” was used for annotations, and top 10 GO terms having statistically significant *P* values from the resulting functional analysis for each group are listed in Table 2. From this table, it can be seen that the GO terms that are enriched in BvL579 glycogenes represent functions necessary for response to wounding, inflammatory response, and blood coagulation (Table 2(a)). The enriched GO terms in BvM501 include functions related to various metabolic (glycoprotein, glycosaminoglycan, sphingolipid, etc.) processes (Table 2(b)). Similarly, functions related to protein maturation, inflammatory response, and complement activation are enriched in LvM442 samples (Table 2(c)).

In addition to DAVID functional analysis, we also identified the biological pathways of BvL579, BvM501, and LvM442 DEGGs annotated in the present study. FASTA formatted amino acid sequences of DEGGs in these three sets were fed into the KAAS for prediction of distinct pathways. A total of 210 pathways were predicted for BvL579, whereas 193 and 198 pathways were predicted for BvM501 and LvM442, respectively. The top 10 KEGG pathways for each of the three categories are shown in Table 3 and a complete list of all pathways is provided in Supplementary Table S1 (Supplementary Material is available online at http://dx.doi.org/10.1155/2014/837365). From these tables, it can be observed that the majority of the DEGGs were found to be associated with important biological processes, many being classified in signaling (e.g., PI3K-Akt and Rap1 signaling) pathways, cytokine-cytokine receptor interaction, or ECM-receptor interaction or being involved in adhesion related functions.

### 3.3. Interaction Network Construction and Identification of Hub Genes

**Figure 2:**
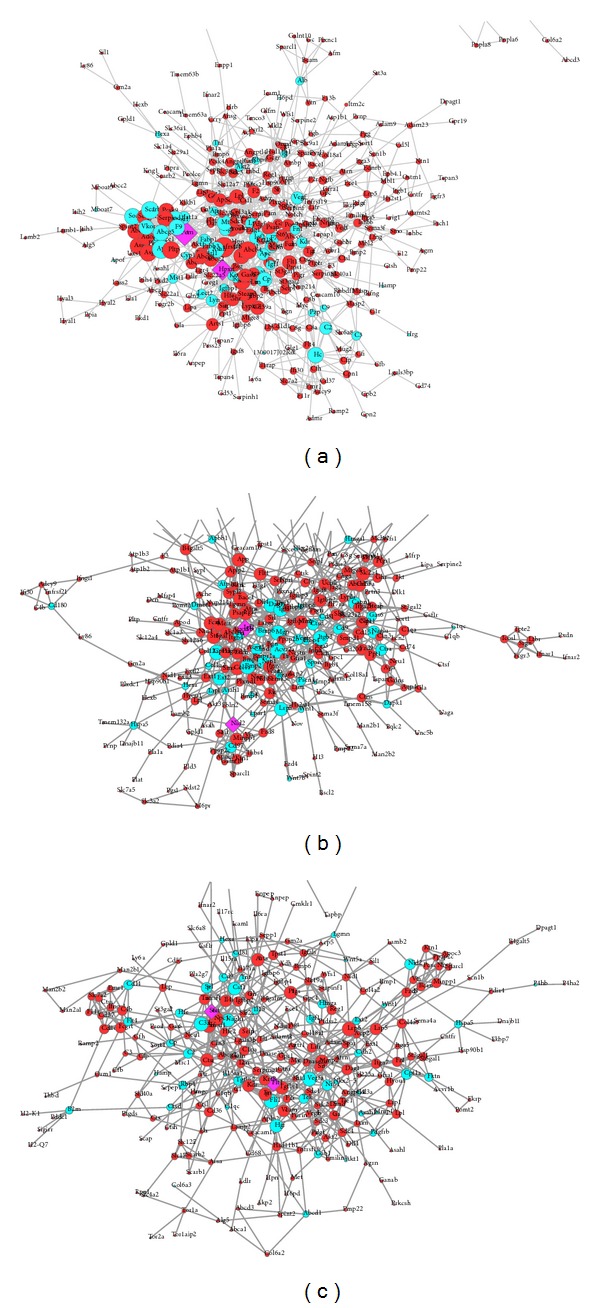
Interaction networks between DEGGs and additional related genes: (a) BvL579, (b) BvM501, and (c) LvM442. In each of these networks, red indicates a DEGG whereas the GeneMANIA predicted genes are shown in cyan. The top two hubs are shown as purple diamonds. The size of the nodes represents the node degree.

DEGGs were mapped to the GeneMANIA to investigate how these genes interact with each other and additional genes that are related to a set of query genes by using a very large set of functional interaction data [30]. The genes showing significant interactions with weights higher than 0.1 were selected only for the network analysis. By integrating these relationships, a network between DEGGs and additional related genes was constructed for all the three groups, namely, BvL579, BvM501, and LvM442 (Figure 2). A GeneMANIA network analysis for interactions among the glycogene products suggested enrichment of GO terms related to positive regulation of locomotion, cell motility, and migration for BvL579 (Table 4(a)), as well as LvM442 samples (Table 4(c)). On the contrary, the processes related to extracellular matrix were overrepresented in BvM501 samples (Table 4(b)).

The three interaction networks created from GeneMANIA were then exported to Cytoscape 2.8.2, a bioinformatics package for biological network visualization and data integration [31]. The initial network for BvL579 consists of 395 nodes and 1149 edges which were further filtered to 395 nodes and 1109 edges by removing duplicate edges (Figure 2(a)). In case of BvM501, the initial network consists of 352 nodes and 794 edges, whereas the filtered network has 352 nodes and 748 edges (Figure 2(b)). Similarly, the initial network for LvM442 network has 307 nodes and 695 edges, while the final network is represented by 307 nodes and 653 edges (Figure 2(c)). All the genes in the network are represented by circles and the interactions between them are represented as edges. Additionally, each query gene is shown in red whereas the additional related genes predicted by GeneMANIA are shown in cyan (Figure 2).

**Figure 3:**
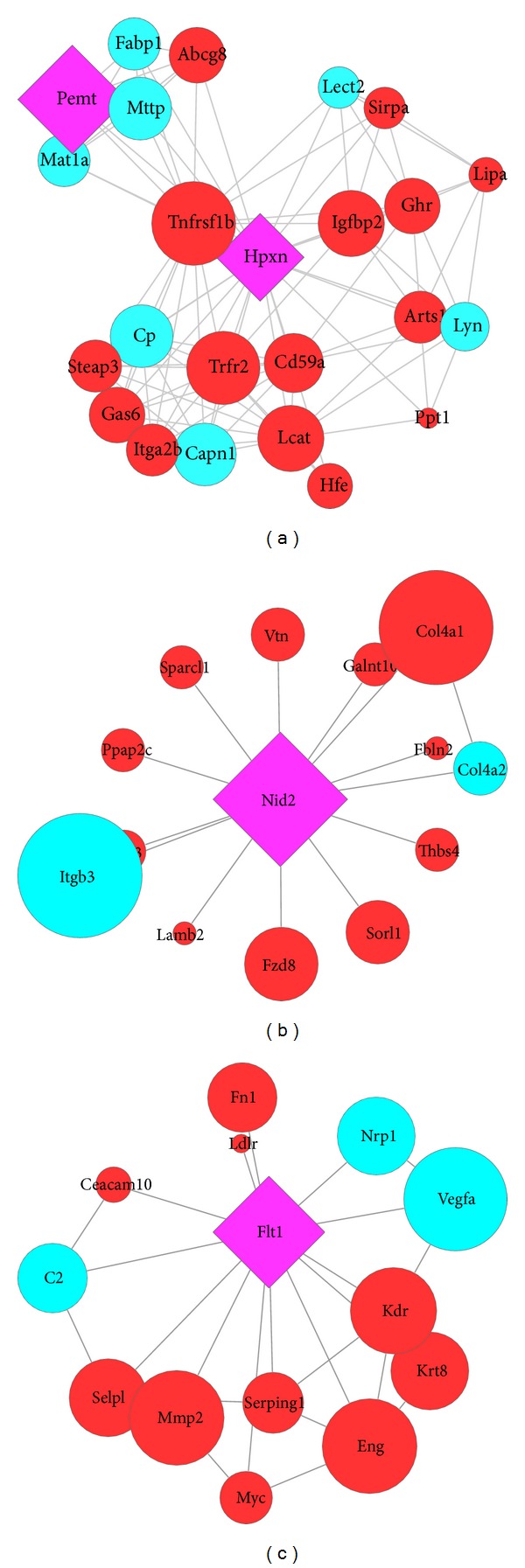
Top glycogenes as hubs and their first neighbors. (a)* HPXN* gene as hubs in BvL579 interaction network. (b)* NID2* as hub gene in BvM501 interaction network. (c)* FLT1* gene as hub in LvM442 interaction network.

The node degree was then calculated for all the nodes in each network by using Network Analyzer plugin of Cytoscape. The higher the node degree, the more important the gene was, and the gene was denoted as a hub gene. The genes with highest node degree include* PEMT* (BvL579) and* IGf2* (BvM501), having node degree of 29 and 14, respectively. Both* PEMT* and* IGF2* were predicted by GeneMANIA as related genes in their respective networks and do not represent the glycogenes. However, the genes with second highest node degrees of 23 and 13 are represented by glycogenes* HPXN* and* NID2* in BvL579 and BvM501 networks (Figures 3(a) and 3(b)). Additionally,* NID2* was also predicted by GeneMANIA as a related gene in BvM501 network because it was removed from the query list as it showed a fold change of less than 2 in our analysis. In LvM442 network, the top node degree genes are* STAT6*, and the glycogene* FLT1 *(Figure 3(c)), both having the node degree of 13.

### 3.4. Community Analysis and Functional Annotation of the Detected Modules

**Figure 4:**
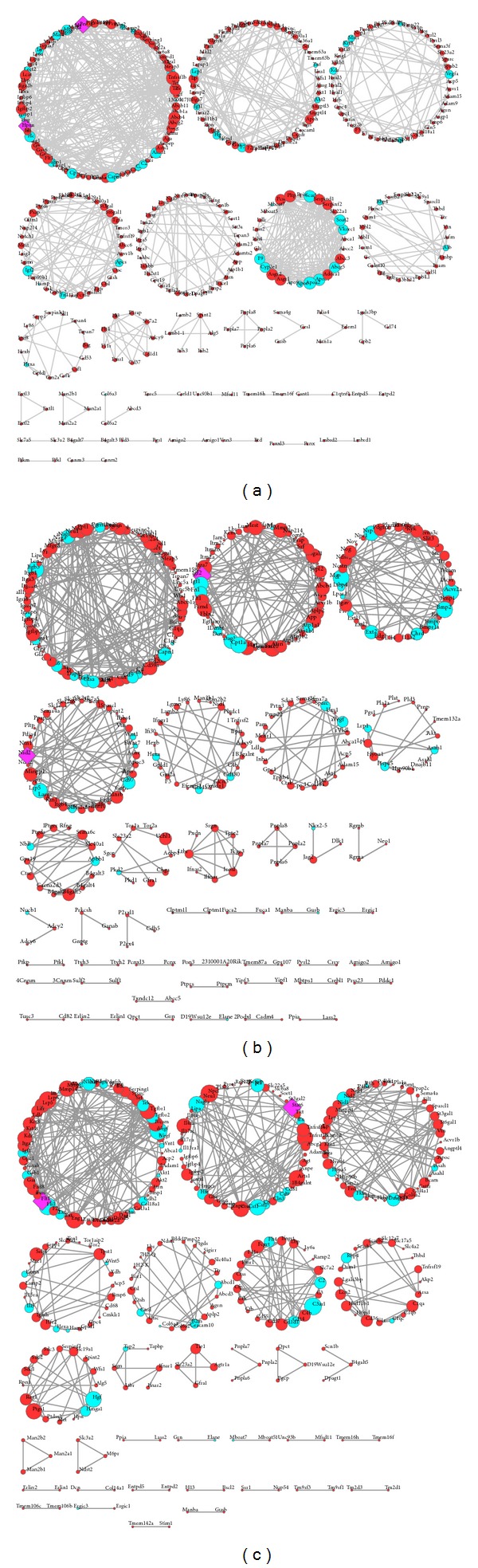
Communities generated by fast greedy (GLay) clustering algorithm are shown. Clusters for (a) BvL579 network, (b) BvM501 network, and (c) LvM442 network are shown. In each community, DEGG nodes are represented by red circles whereas the cyan nodes represent GeneMANIA predicted genes. Hub genes are shown as purple diamonds.

In order to identify the biologically related genes in the three networks, we employed Fast Greedy community-structure identification algorithm, implemented in the Cytoscape plugin GLay (http://brainarray.mbni.med.umich.edu/sugang/glay/) [29] to identify functional modules, 31 (Figure 4(a)), 40 (Figure 4(b)), and 31 (Figure 4(c)) clusters were detected for BvL579, BvM501, and LvM442 networks, respectively. However, only those communities were selected for enrichment analyses which have at least 10 nodes. Therefore, based on this criterion, 8, 9, and 8 communities for BvL579, BvM501, and LvM442 networks were finally analyzed for overrepresentation of GO terms.

To biologically categorize these clusters, DAVID functional analysis tool was used to classify the genes in each module and observed the enrichment of GO term “Biological Process” in all the selected modules. The enrichment analysis for the modules for three networks is described as follows.

*BvL579.* The top 10 statistically significant enriched GO terms for DEGGs in all 8 clusters for BvL579 community analysis are summarized in Table 5(a). The most statistically significant GO terms that were enriched in cluster 1 were related to immune response, complement activation, and protein maturation. Clusters 2, 3, and 7 show enrichment for GO terms response to wounding, blood coagulation, and hemostasis. Clusters 2 and 3 also show overrepresentation of terms such as blood vessel development and vasculature development. The GO terms of cluster 4 were mostly related to hair cycle, development of epidermis, and ectoderm. The GO functions of cluster 5 were most related to localization, motility and migration of cells, and regulation of cell proliferation. Cluster 6 was significantly enriched with functions related to homeostasis, transport, and metabolism of cholesterol, and lipid as well as sterol. Cluster 8 showed overrepresentation of functional terms related to the metabolism of glycosphingolipid, glycolipid, sphingolipid, and ganglioside.

*BvM501.* The top 10 statistically significant enriched GO terms for DEGGs in all 9 clusters for BvM501 community analysis are summarized in (Table 5(b)). Cluster 1 of this network shows overrepresentation of functions related to regulation of cell growth, protein maturation, and immune response. Cluster 2 is enriched in GO terms related to proteoglycan metabolic process as well as the development of blood vessel and vasculature. Cluster 3 is enriched in the functions related to the development of bone, cartilage, and skeletal system. The GO terms that are overrepresented in cluster 4 show enrichment for functions related to adhesion, such as biological adhesion, cell adhesion, cell-substrate adhesion, and cell-matrix adhesion. The processes related to localization, motility, and migration of cells are also overrepresented in cluster 4. Cluster 5 showed overrepresentation of functional terms related to the metabolism of glycosphingolipid, glycolipid, sphingolipid, and ganglioside. Additionally, the functions related to the metabolism of lipid and oligosaccharides are also enriched in this group. In cluster 6, functions related to homeostasis, remodelling, and regulation of transport are overrepresented. Response to hypoxia and oxygen levels, apoptosis, and metabolic processes such as phospholipid and organophosphate metabolic process are enriched in cluster 7. Similar to cluster 4, the GO terms that are overrepresented in Cluster 9 show enrichment for functions related to biological adhesion. Protein folding and metal ion transport are the other important enriched functions present in this cluster. Cluster 8 did not yield the enrichment of any statistically significant GO category.

*LvM442.* Table 5(c) summarizes the top 10 statistically significant enriched GO terms for DEGGs in all 8 clusters for LvM442 community analysis. The GO terms related to development and morphogenesis of blood vessels, vasculature, and tube are enriched in cluster 1. Cluster 2 shows enrichment of functions related to various types of responses, such as response to wounding, external stimulus, and defense as well as inflammatory response. Other enriched functional groups in this cluster are regulation of cell growth, proliferation, and cell activation. One of the overrepresented functional groups in cluster 3 shows enrichment for functions related to biological adhesion, lipid catabolic process, and 4-hydroxyproline metabolic process. Cluster 4 represents the enrichment of functions related to protein folding and bone remodeling and resorption. Cluster 5 shows overrepresentation of GO terms such as regulation of T cell mediated cytotoxicity or immunity, regulation of leukocyte mediated cytotoxicity, and regulation of cell killing. Clusters 6 and 7 are enriched in functions related to immune or inflammatory response and complement activation. Finally, cluster 8 exhibited overrepresentation of signaling pathways such as hepatocyte growth factor receptor signaling and mesenchymal-epithelial cell signaling, in addition to processes concerning MAP kinase activity.

## 4. Discussion

The current study offers the first thorough insight into the glycogene analysis of brain, muscle, and liver tissues from mouse RNA-Seq data. Understanding the structure and function of these glycogenes is essential for studying the development of various tissues as well as their functional roles. Most of the serum glycoproteins originates from liver suggesting that liver diseases associated with aberrant glycosylation can be reflected by the changes in serum glycoproteins [32]. Recently, the significance of glycomics of central nervous system (CNS) to identify potential glycobiomarkers in neurological diseases [33] and alterations in brain glycoproteins resulting from the aging process was shown [34]. Recent studies in the glycomics field also offered insights into the biological significance of the glycome in the pathogenesis of diseases in humans [32]. Additionally, many studies have highlighted the significance of glycoconjugates during skeletal muscle development [7, 23, 35].

With the evolution of bioinformatics during the past decades, molecular target discovery and targeted therapeutics have become a critical remedial treatment for diseases [36]. In this work, we screened DEGGs between brain, muscle, and liver tissues using RNA-Seq data and performed functional and pathway enrichment analysis using DAVID and KEGG tools. The most significant enriched GO terms for BvL579 are processes related to response to wounding or inflammatory response. These processes are represented by genes that play a prominent roles in innate immunity such as* Mannan-binding lectin serine protease 1* (*MASP1*),* Mannan-binding lectin serine protease 2* (*MASP2*),* Mannose-binding protein A* (*MBL1*), and* Mannose-binding protein C* (*MBL2*) [37, 38]. BvM501 is enriched in functions related to glycoprotein metabolic processes and includes genes that code for various transferases such as N-acetylglucosamine-1-phosphotransferase subunit gamma (GNPTG) [39], Beta-galactoside alpha-2,6-sialyltransferase 1 (ST6GAL1) [40], and Beta-1,3-galactosyltransferase 6 (B3GALT6) [41]. Similarly, the top statistically significant enriched processes in LvM442 are related to protein maturation or processing and are represented by genes such as* Battenin* (*CLN3*) and* Methionine aminopeptidase 2* (*METAP2*). The CLN3 protein is involved in the late endosomal/lysosomal membrane transport [42], whereas METAP2 protein catalyzes the hydrolytic cleavage of the N-terminal methionine from newly synthesized polypeptides [43].

Additionally, three interaction networks of DEGGs were constructed for BvL579, BvM501, and LvM442 samples, and node degrees of genes were calculated.* HPXN*,* NID2*, and* FLT1 *were the glycogenes with the highest degree in BvL579, BvM501, and LvM442 networks, respectively. However, these genes represent the second highest node degree in their respective networks as genes (*PEMT*,* IGF2*, and* STAT6*) with top node degree that do not code for glycoproteins.

HPXN (~60 kDa glycoprotein) is mainly synthesized by liver cells [44], secreted to the plasma where it binds free heme or hemin and inhibits its role in free radical reactions [45]. HPXN is also reported to be expressed by the cells of immune systems, ganglionic and photoreceptor cells of the retina, cells of the peripheral nervous system, and the mesangial cells of kidney [46–48]. Recently, it was shown that HPX protein is also present in various regions of mouse brain [49]. NID2 is homologous to another member of the nidogen family, NID1, and both are found in all basement membranes (BMs) [50]. Both nidogens have a similar distribution in various organs during development; however, in adult tissues nidogen-2 distribution becomes more confined [51–53]. Additionally, these nidogens show a broad range of interacting partners including other BM proteins such as laminin, collagen IV, and perlecan [51, 53–55]. They are involved in various functions including the regulation of cell attachment [56], neutrophil chemotaxis [57], trophoblast outgrowth [58], angiogenesis [59], osteoblast, and myogenic differentiation [60, 61]. Vascular endothelial growth factors (VEGFs) constitute a family of six polypeptides (VEGF-A, -B, -C, -D, -E, and PlGF) that regulate blood and lymphatic vessel development [62]. VEGF signaling occurs by binding to various cellular receptors such as VEGFR1 (FLT1), VEGFR2 (FLK1), and VEGFR3 (FLT4) [63, 64] and neuropilin-1 [65] and -2 (NRP1 and NRP2) [66] and heparan sulfate proteoglycans (HSPG) [67]. FLT1 and FLK1 are closely related receptor tyrosine kinases and both share common and specific ligands [68]. FLT1 has weaker kinase activity than FLK1 [68]; however, FLT1 is essential for normal development and angiogenesis as reported in previous FLT1 null mutant mice studies [69–71].

Recognizing the structure and function of biological networks is indispensable for the investigation of biological processes. In this work, we identified 8, 9, and 8 functional modules or communities for BvL579, BvM501, and LvM442 networks using fast greedy algorithm implemented as GLAY [29], plugin for cytoscape. Furthermore, the function of each module in all the three networks was explored by using functional annotation tool DAVID. Our analysis shows that the modules that are common in BvL579 and LvM442 networks show enrichment for processes related to immune and inflammatory response and response to wounding. Liver is known to play an important role in innate immunity, an important primary line of defense against infection [72]. Additionally, Kupffer cells in the liver are one of the earliest to be affected by bacterial or sterile insults and add to the inflammatory response [73]. This sterile inflammation can be responsible for liver injury; it may also play a role in liver repair [72]. In case of the modules of BvM501 and LvM442 networks, the enrichment of processes related to cell adhesion, proliferation, and regulation of cell growth is observed. The expression of myogenic regulatory factors (MRFs) describes the different stages of skeletal muscle development that includes myoblast proliferation, cell-cycle exit, cell fusion, and the maturation of myotubes to form myofibers [23]. Cell adhesion molecules such as cadherins are glycoproteins that mediate homotypic cell-cell adhesion through their extracellular domain [74], and this cadherin-dependent adhesion is necessary for diverse types of cellular functions [75]. Finally, glycosphingolipid or glycolipid metabolic processes show overrepresentation in modules of BvL579 and BvM501 networks. Glycosphingolipids are universally expressed in all vertebrate cells as well as body fluids, but they are richer in the nervous system [76]. Previous studies have established the role of glycosphingolipids in the development of the brain among various species [77, 78].

## 5. Conclusion

Overall, in this work we have used a systems biology approach to identify the DEGGs, their functional enrichment, and identified potential hub genes by constructing three glycogene interaction networks. We also identified functional modules in these networks and indicate the significance of immune system related processes in liver, and glycosphingolipid metabolic processes in the development of brain. Similarly, we observe that the processes such as cell proliferation, adhesion, and growth are important for muscle development. The findings deduced from the current work are in consistence with the previous studies. Experimental validation will be required to confirm the predictions made in this study that will establish the role of predicted hubs as well as enriched functional processes in these tissues.

## Supplementary Material

Supplementary Table S1: List of all KEGG pathways reported for different tissue groups. Each sheet contains the list of KEGG pathways and the number of mapped genes in brain versus liver (BvL579), brain versus muscle (BvM501), and liver versus muscle (LvM442) groups.

## Figures and Tables

**Table 1a:** (a) Total number of genes in each sample before and after filtering. Each tissue sample consists of 33598 genes that were filtered by removing all those genes that showed RPKM value < 1

	Data filtering	
Tissue	Total number of genes	Total number of genes after filtering
Brain	33598	15,237
Liver	33598	11,920
Muscle	33598	12,202

**Table 1b:** (b) Total number of differentially expressed genes between different tissues

	Genes commonly expressed between different tissues		
Tissue	Number of expressed genes	Number of differentially expressed genes (DEGs) (≥2 fold change)	Number of differentially expressed glycogenes (DEGGs) (≥2 fold change)
Brain versus liver	10,653	5228	579
Brain versus muscle	10,820	5283	501
Liver versus muscle	9,833	3840	442

**Table 2a:** (a) BvL579

GO Term	Total number of genes	*P* value
GO:0009611~response to wounding	53	2.98*E − *23
GO:0002526~acute inflammatory response	23	4.83*E − *16
GO:0006954~inflammatory response	35	1.43*E − *15
GO:0051604~protein maturation	23	2.51*E − *14
GO:0007596~blood coagulation	20	5.40*E − *14
GO:0050817~coagulation	20	5.40*E − *14
GO:0007599~hemostasis	20	7.17*E − *14
GO:0006952~defense response	45	7.57*E − *13
GO:0050878~regulation of body fluid levels	20	5.84*E − *12
GO:0016485~protein processing	20	7.21*E − *12

**Table 2b:** (b) BvM501

GO Term	Total number of genes	*P* value
GO:0009100~glycoprotein metabolic process	30	1.76*E − *18
GO:0009101~glycoprotein biosynthetic process	23	8.1*E − *14
GO:0070085~glycosylation	17	6.09*E − *10
GO:0006486~protein amino acid glycosylation	17	6.09*E − *10
GO:0043413~biopolymer glycosylation	17	6.09*E − *10
GO:0030203~glycosaminoglycan metabolic process	11	3.1*E − *08
GO:0006665~sphingolipid metabolic process	12	2.79*E − *07
GO:0006643~membrane lipid metabolic process	12	3.9*E − *07
GO:0006022~aminoglycan metabolic process	11	4.45*E − *07
GO:0051604~protein maturation	14	6.1*E − *07

**Table 2c:** (c) LvM442

GO Term	Total number of genes	*P* value
GO:0051604~protein maturation	20	3.17*E − *13
GO:0016485~protein processing	19	1.12*E − *12
GO:0009100~glycoprotein metabolic process	23	1.63*E − *12
GO:0006954~inflammatory response	27	6.69*E − *12
GO:0009611~response to wounding	33	1.07*E − *11
GO:0002526~acute inflammatory response	17	2.53*E − *11
GO:0009101~glycoprotein biosynthetic process	19	1.14*E − *10
GO:0051605~protein maturation by peptide bond cleavage	15	1.33*E − *10
GO:0006956~complement activation	11	4.25*E − *09
GO:0002541~activation of plasma proteins involved in acute inflammatory response	11	4.25*E − *09

**Table 3a:** (a) BvL579

Pathway name	Number of mapped Genes
04151 PI3K-Akt signaling pathway	34
04610 Complement and coagulation cascades	34
04142 Lysosome	31
04060 Cytokine-cytokine receptor interaction	23
05200 Pathways in cancer	21
04015 Rap1 signaling pathway	18
04510 Focal adhesion	18
05205 Proteoglycans in cancer	18
04514 Cell adhesion molecules CAMs	16
04976 Bile secretion	16

**Table 3b:** (b) BvM501

Pathway name	Number of mapped Genes
04142 Lysosome	29
04151 PI3K-Akt signaling pathway	24
04060 Cytokine-cytokine receptor interaction	19
04514 Cell adhesion molecules CAMs	19
05200 Pathways in cancer	17
04141 Protein processing in endoplasmic reticulum	15
04510 Focal adhesion	15
05166 HTLV-I infection	13
04512 ECM-receptor interaction	12
04610 Complement and coagulation cascades	12

**Table 3c:** (c) LvM442

Pathway name	Number of mapped Genes
04142 Lysosome	33
04151 PI3K-Akt signaling pathway	26
04060 Cytokine-cytokine receptor interaction	23
04141 Protein processing in endoplasmic reticulum	17
05205 Proteoglycans in cancer	17
04610 Complement and coagulation cascades	15
04514 Cell adhesion molecules CAMs	14
04510 Focal adhesion	14
04512 ECM-receptor interaction	13
04145 Phagosome	13

**Table 4a:** (a) BvL579

GO id	Description	*q*-value
GO:0040017	positive regulation of locomotion	1.83*E − *23
GO:2000147	positive regulation of cell motility	1.83*E − *23
GO:0030335	positive regulation of cell migration	3.40*E − *23
GO:0051272	positive regulation of cellular component movement	3.40*E − *23
GO:0050817	coagulation	1.94*E − *20
GO:0010876	lipid localization	3.80*E − *20
GO:0007596	blood coagulation	4.54*E − *20
GO:0007599	hemostasis	7.56*E − *20
GO:0009897	external side of plasma membrane	2.15*E − *18
GO:0000323	lytic vacuole	1.05*E − *17

**Table 4b:** (b) BvM501

GO id	Description	*q*-value
GO:0031012	extracellular matrix	2.43*E − *23
GO:0005578	proteinaceous extracellular matrix	1.37*E − *17
GO:0009100	glycoprotein metabolic process	1.99*E − *16
GO:0000323	lytic vacuole	2.87*E − *16
GO:0005764	lysosome	2.87*E − *16
GO:0044420	extracellular matrix part	3.18*E − *15
GO:0005604	basement membrane	1.20*E − *14
GO:0005773	vacuole	4.56*E − *14
GO:0030335	positive regulation of cell migration	5.17*E − *12
GO:0005178	integrin binding	5.17*E − *12

**Table 4c:** (c) LvM442

GO id	Description	*q*-value
GO:0000323	lytic vacuole	1.09*E − *20
GO:0005764	lysosome	1.09*E − *20
GO:0051272	positive regulation of cellular component movement	8.78*E − *20
GO:0030335	positive regulation of cell migration	1.04*E − *19
GO:2000147	positive regulation of cell motility	1.79*E − *19
GO:0040017	positive regulation of locomotion	6.43*E − *19
GO:0005773	vacuole	1.70*E − *18
GO:0019838	growth factor binding	2.80*E − *18
GO:0009100	glycoprotein metabolic process	2.11*E − *15
GO:0031012	extracellular matrix	1.48*E − *14

**Table 5a:** (a) BvL579

Cluster number	Term	*P* value
1	GO:0002541~activation of plasma proteins involved in acute inflammatory response	5.02*E − *22
	GO:0006956~complement activation	5.02*E − *22
	GO:0002526~acute inflammatory response	2.26*E − *20
	GO:0006959~humoral immune response	2.25*E − *19
	GO:0051604~protein maturation	3.43*E − *19
	GO:0051605~protein maturation by peptide bond cleavage	3.19*E − *18
	GO:0002253~activation of immune response	3.20*E − *18
	GO:0016485~protein processing	6.27*E − *18
	GO:0006957~complement activation, alternative pathway	8.91*E − *18
	GO:0006954~inflammatory response	1.44*E − *17

2	GO:0051094~positive regulation of developmental process	5.33*E − *05
	GO:0042592~homeostatic process	4.25*E − *04
	GO:0009611~response to wounding	7.29*E − *04
	GO:0001568~blood vessel development	9.88*E − *04
	GO:0001944~vasculature development	0.001101764
	GO:0050817~coagulation	0.001394123
	GO:0007596~blood coagulation	0.001394123
	GO:0007599~hemostasis	0.001452434
	GO:0050878~regulation of body fluid levels	0.002774109
	GO:0007169~transmembrane receptor protein tyrosine kinase signaling pathway	0.003100357

3	GO:0050817~coagulation	2.74*E − *11
	GO:0007596~blood coagulation	2.74*E − *11
	GO:0007599~hemostasis	3.08*E − *11
	GO:0050878~regulation of body fluid levels	1.97*E − *10
	GO:0042060~wound healing	1.26*E − *09
	GO:0009611~response to wounding	5.75*E − *08
	GO:0007167~enzyme linked receptor protein signaling pathway	9.33*E − *08
	GO:0001568~blood vessel development	5.52*E − *07
	GO:0001944~vasculature development	6.64*E − *07
	GO:0001525~angiogenesis	4.71*E − *05

4	GO:0022404~molting cycle process	2.16*E − *04
	GO:0022405~hair cycle process	2.16*E − *04
	GO:0001942~hair follicle development	2.16*E − *04
	GO:0042633~hair cycle	2.42*E − *04
	GO:0042303~molting cycle	2.42*E − *04
	GO:0008544~epidermis development	0.003102493
	GO:0007398~ectoderm development	0.00369522
	GO:0050678~regulation of epithelial cell proliferation	0.009595347
	GO:0048732~gland development	0.01092817
	GO:0007173~epidermal growth factor receptor signaling pathway	0.025611634

5	GO:0007166~cell surface receptor linked signal transduction	1.34*E − *05
	GO:0016477~cell migration	2.69*E − *05
	GO:0042127~regulation of cell proliferation	3.44*E − *05
	GO:0051674~localization of cell	4.88*E − *05
	GO:0048870~cell motility	4.88*E − *05
	GO:0006928~cell motion	6.35*E − *05
	GO:0008284~positive regulation of cell proliferation	2.52*E − *04
	GO:0007229~integrin-mediated signaling pathway	4.87*E − *04
	GO:0040007~growth	6.74*E − *04
	GO:0007507~heart development	0.001229375

6	GO:0055088~lipid homeostasis	2.16*E − *16
	GO:0042632~cholesterol homeostasis	2.64*E − *15
	GO:0055092~sterol homeostasis	2.64*E − *15
	GO:0030301~cholesterol transport	3.97*E − *13
	GO:0015918~sterol transport	3.97*E − *13
	GO:0033344~cholesterol efflux	7.23*E − *13
	GO:0006869~lipid transport	3.89*E − *12
	GO:0008203~cholesterol metabolic process	4.74*E − *12
	GO:0010876~lipid localization	8.01*E − *12
	GO:0016125~sterol metabolic process	1.02*E − *11

7	GO:0009611~response to wounding	3.88*E − *07
	GO:0007596~blood coagulation	2.94*E − *06
	GO:0050817~coagulation	2.94*E − *06
	GO:0007599~hemostasis	3.11*E − *06
	GO:0050878~regulation of body fluid levels	7.69*E − *06
	GO:0042060~wound healing	1.91*E − *05
	GO:0051818~disruption of cells of other organism during symbiotic interaction	3.08*E − *05
	GO:0051851~modification by host of symbiont morphology or physiology	3.08*E − *05
	GO:0051883~killing of cells in other organism during symbiotic interaction	3.08*E − *05
	GO:0031640~killing of cells of another organism	4.30*E − *05

8	GO:0006689~ganglioside catabolic process	5.11*E − *06
	GO:0046479~glycosphingolipid catabolic process	1.79*E − *05
	GO:0019377~glycolipid catabolic process	1.79*E − *05
	GO:0001573~ganglioside metabolic process	2.38*E − *05
	GO:0046466~membrane lipid catabolic process	1.15*E − *04
	GO:0030149~sphingolipid catabolic process	1.15*E − *04
	GO:0019915~lipid storage	1.29*E − *04
	GO:0006687~glycosphingolipid metabolic process	2.73*E − *04
	GO:0006664~glycolipid metabolic process	3.90*E − *04
	GO:0050885~neuromuscular process controlling balance	4.98*E − *04

**Table 5b:** (b) BvM501

Cluster number	Term	*P* value
1	GO:0040008~regulation of growth	3.73*E − *08
	GO:0051604~protein maturation	7.27*E − *08
	GO:0001558~regulation of cell growth	1.29*E − *06
	GO:0016485~protein processing	2.32*E − *05
	GO:0016044~membrane organization	7.38*E − *05
	GO:0016064~immunoglobulin mediated immune response	9.61*E − *05
	GO:0051605~protein maturation by peptide bond cleavage	1.09*E − *04
	GO:0019724~B cell mediated immunity	1.09*E − *04
	GO:0002684~positive regulation of immune system process	1.29*E − *04
	GO:0006958~complement activation, classical pathway	2.00*E − *04

2	GO:0009100~glycoprotein metabolic process	2.16*E − *06
	GO:0050654~chondroitin sulfate proteoglycan metabolic process	9.34*E − *06
	GO:0006029~proteoglycan metabolic process	8.82*E − *05
	GO:0048754~branching morphogenesis of a tube	1.02*E − *04
	GO:0019800~peptide cross-linking via chondroitin 4-sulfate glycosaminoglycan	1.89*E − *04
	GO:0001763~morphogenesis of a branching structure	3.20*E − *04
	GO:0001568~blood vessel development	4.29*E − *04
	GO:0001944~vasculature development	4.79*E − *04
	GO:0030204~chondroitin sulfate metabolic process	6.09*E − *04
	GO:0006813~potassium ion transport	8.13*E − *04

3	GO:0001501~skeletal system development	1.07*E − *16
	GO:0030509~BMP signaling pathway	6.34*E − *14
	GO:0001503~ossification	4.02*E − *12
	GO:0060348~bone development	7.43*E − *12
	GO:0030500~regulation of bone mineralization	6.08*E − *11
	GO:0007178~transmembrane receptor protein serine/threonine kinase signaling pathway	7.75*E − *11
	GO:0070167~regulation of biomineral formation	9.25*E − *11
	GO:0007167~enzyme linked receptor protein signaling pathway	1.84*E − *10
	GO:0007369~gastrulation	2.22*E − *10
	GO:0051216~cartilage development	3.31*E − *10

4	GO:0006928~cell motion	9.88*E − *05
	GO:0016055~Wnt receptor signaling pathway	2.59*E − *04
	GO:0016477~cell migration	4.37*E − *04
	GO:0048870~cell motility	7.09*E − *04
	GO:0051674~localization of cell	7.09*E − *04
	GO:0007160~cell-matrix adhesion	0.001189285
	GO:0009100~glycoprotein metabolic process	0.001250564
	GO:0031589~cell-substrate adhesion	0.001570006
	GO:0007155~cell adhesion	0.005368157
	GO:0022610~biological adhesion	0.005405426

5	GO:0006689~ganglioside catabolic process	1.66*E − *05
	GO:0019377~glycolipid catabolic process	5.78*E − *05
	GO:0046479~glycosphingolipid catabolic process	5.78*E − *05
	GO:0001573~ganglioside metabolic process	7.69*E − *05
	GO:0009611~response to wounding	2.01*E − *04
	GO:0046466~membrane lipid catabolic process	3.70*E − *04
	GO:0030149~sphingolipid catabolic process	3.70*E − *04
	GO:0019915~lipid storage	4.16*E − *04
	GO:0006687~glycosphingolipid metabolic process	8.77*E − *04
	GO:0009311~oligosaccharide metabolic process	0.001247784

6	GO:0042592~homeostatic process	6.03*E − *06
	GO:0043270~positive regulation of ion transport	0.001725638
	GO:0051270~regulation of cell motion	0.002725255
	GO:0048771~tissue remodeling	0.003048492
	GO:0001894~tissue homeostasis	0.003842174
	GO:0051050~positive regulation of transport	0.004095371
	GO:0007169~transmembrane receptor protein tyrosine kinase signaling pathway	0.004147057
	GO:0032869~cellular response to insulin stimulus	0.004461779
	GO:0042127~regulation of cell proliferation	0.004903945
	GO:0010765~positive regulation of sodium ion transport	0.005901212

7	GO:0001666~response to hypoxia	0.006023043
	GO:0070482~response to oxygen levels	0.006648281
	GO:0016042~lipid catabolic process	0.009864913
	GO:0006644~phospholipid metabolic process	0.011805904
	GO:0019637~organophosphate metabolic process	0.013020542
	GO:0006916~anti-apoptosis	0.013774773
	GO:0043066~negative regulation of apoptosis	0.037899056
	GO:0043069~negative regulation of programmed cell death	0.038883095
	GO:0060548~negative regulation of cell death	0.03908111

8	NA	NA

9	GO:0007160~cell-matrix adhesion	0.001495186
	GO:0031589~cell-substrate adhesion	0.001809131
	GO:0051085~chaperone mediated protein folding requiring cofactor	0.006635187
	GO:0051084~“de novo” posttranslational protein folding	0.008618097
	GO:0007155~cell adhesion	0.00916406
	GO:0022610~biological adhesion	0.009200307
	GO:0050982~detection of mechanical stimulus	0.010597488
	GO:0006458~“de novo” protein folding	0.010597488
	GO:0007259~JAK-STAT cascade	0.025656487
	GO:0030001~metal ion transport	0.036163834
	GO:0030001~metal ion transport	0.036163834

**Table 5c:** (c) LvM442

Cluster number	Term	*P* value
1	GO:0001568~blood vessel development	1.06*E − *18
	GO:0001944~vasculature development	1.64*E − *18
	GO:0048514~blood vessel morphogenesis	8.45*E − *16
	GO:0001525~angiogenesis	3.48*E − *12
	GO:0048754~branching morphogenesis of a tube	9.17*E − *11
	GO:0007167~enzyme linked receptor protein signaling pathway	5.50*E − *10
	GO:0035239~tube morphogenesis	1.14*E − *09
	GO:0001763~morphogenesis of a branching structure	1.32*E − *09
	GO:0035295~tube development	5.53*E − *09
	GO:0001569~patterning of blood vessels	3.29*E − *08

2	GO:0040008~regulation of growth	4.27*E − *07
	GO:0009611~response to wounding	4.18*E − *06
	GO:0006954~inflammatory response	3.42*E − *05
	GO:0032101~regulation of response to external stimulus	1.89*E − *04
	GO:0048585~negative regulation of response to stimulus	7.82*E − *04
	GO:0006952~defense response	0.001399518
	GO:0001558~regulation of cell growth	0.002134607
	GO:0050867~positive regulation of cell activation	0.002784166
	GO:0032102~negative regulation of response to external stimulus	0.004518678
	GO:0008283~cell proliferation	0.004842417

3	GO:0007160~cell-matrix adhesion	7.84*E − *05
	GO:0031589~cell-substrate adhesion	1.14*E − *04
	GO:0044242~cellular lipid catabolic process	9.80*E − *04
	GO:0016042~lipid catabolic process	9.93*E − *04
	GO:0051346~negative regulation of hydrolase activity	0.006807864
	GO:0008218~bioluminescence	0.007742186
	GO:0018401~peptidyl-proline hydroxylation to 4-hydroxy-L-proline	0.007742186
	GO:0019471~4-hydroxyproline metabolic process	0.007742186
	GO:0009101~glycoprotein biosynthetic process	0.007780584
	GO:0007155~cell adhesion	0.00843311

4	GO:0001501~skeletal system development	7.15*E − *04
	GO:0006955~immune response	0.011579
	GO:0051085~chaperone mediated protein folding requiring cofactor	0.013230724
	GO:0051084~“de novo” posttranslational protein folding	0.01716759
	GO:0045453~bone resorption	0.019783921
	GO:0006458~“de novo” protein folding	0.021089619
	GO:0046849~bone remodeling	0.027593503
	GO:0006952~defense response	0.045862312

5	GO:0001916~positive regulation of T cell mediated cytotoxicity	4.08*E − *05
	GO:0001914~regulation of T cell mediated cytotoxicity	6.22*E − *05
	GO:0002711~positive regulation of T cell mediated immunity	8.81*E − *05
	GO:0002474~antigen processing and presentation of peptide antigen via MHC class I	1.53*E − *04
	GO:0002709~regulation of T cell mediated immunity	1.72*E − *04
	GO:0031343~positive regulation of cell killing	2.84*E − *04
	GO:0001912~positive regulation of leukocyte mediated cytotoxicity	2.84*E − *04
	GO:0001910~regulation of leukocyte mediated cytotoxicity	4.86*E − *04
	GO:0031341~regulation of cell killing	4.86*E − *04
	GO:0002821~positive regulation of adaptive immune response	6.25*E − *04

6	GO:0002526~acute inflammatory response	4.67*E − *14
	GO:0045087~innate immune response	4.65*E − *13
	GO:0002684~positive regulation of immune system process	1.53*E − *12
	GO:0050778~positive regulation of immune response	3.29*E − *12
	GO:0006954~inflammatory response	3.41*E − *12
	GO:0002541~activation of plasma proteins involved in acute inflammatory response	4.05*E − *12
	GO:0006956~complement activation	4.05*E − *12
	GO:0048584~positive regulation of response to stimulus	4.13*E − *11
	GO:0006952~defense response	4.75*E − *11
	GO:0006959~humoral immune response	5.29*E − *11

7	GO:0009611~response to wounding	2.97*E − *06
	GO:0006958~complement activation, classical pathway	7.07*E − *06
	GO:0002526~acute inflammatory response	7.33*E − *06
	GO:0002455~humoral immune response mediated by circulating immunoglobulin	8.69*E − *06
	GO:0006956~complement activation	2.20*E − *05
	GO:0002541~activation of plasma proteins involved in acute inflammatory response	2.36*E − *05
	GO:0016064~immunoglobulin mediated immune response	4.70*E − *05
	GO:0019724~B cell mediated immunity	5.25*E − *05
	GO:0002449~lymphocyte mediated immunity	1.02*E − *04
	GO:0002250~adaptive immune response	1.36*E − *04

8	GO:0051450~myoblast proliferation	0.002206368
	GO:0048012~hepatocyte growth factor receptor signaling pathway	0.002206368
	GO:0060665~regulation of branching involved in salivary gland morphogenesis by mesenchymal-epithelial signaling	0.003674845
	GO:0060638~mesenchymal-epithelial cell signaling	0.004408354
	GO:0060693~regulation of branching involved in salivary gland morphogenesis	0.006605966
	GO:0060688~regulation of morphogenesis of a branching structure	0.018252999
	GO:0001889~liver development	0.031208893
	GO:0000187~activation of MAPK activity	0.036205594
	GO:0043406~positive regulation of MAP kinase activity	0.042595839
