# Supplementary material for: Comparative Analysis of Glycogene Expression in Different Mouse Tissues Using RNA-Seq Data
Source: Int J Genomics. 2014 Jul 9;2014:837365. doi: 10.1155/2014/837365 (PMC4121153; doi:10.1155/2014/837365)
Supplement: Supplementary file 1 — Supplementary Table S1: List of all KEGG pathways reported for different tissue groups. Each sheet contains the list of KEGG pathways and the number of mapped genes in brain versus liver (BvL579), brain versus muscle (BvM501), and liver versus muscle (LvM442) groups. [file 837365.f1.pdf]

Pathway Name	No. of Mapped Genes
04142 Lysosome	33
04151 PI3K-Akt signaling pathway	26
04060 Cytokine-cytokine receptor interaction	23
04141 Protein processing in endoplasmic reticulum	17
05205 Proteoglycans in cancer	17
04610 Complement and coagulation cascades	15
04514 Cell adhesion molecules CAMs	14
04510 Focal adhesion	14
04512 ECM-receptor interaction	13
04145 Phagosome	13
05200 Pathways in cancer	13
04630 Jak-STAT signaling pathway	11
05150 Staphylococcus aureus infection	11
05166 HTLV-I infection	11
00510 N-Glycan biosynthesis	10
04014 Ras signaling pathway	10
04015 Rap1 signaling pathway	10
04640 Hematopoietic cell lineage	10
05152 Tuberculosis	9
00564 Glycerophospholipid metabolism	8
04380 Osteoclast differentiation	8
05322 Systemic lupus erythematosus	8
05133 Pertussis	8
05168 Herpes simplex infection	8
05144 Malaria	8
04144 Endocytosis	7
04670 Leukocyte transendothelial migration	7
05164 Influenza A	7
00561 Glycerolipid metabolism	6
00531 Glycosaminoglycan degradation	6
04668 TNF signaling pathway	6
04066 HIF-1 signaling pathway	6
04976 Bile secretion	6
04974 Protein digestion and absorption	6
05202 Transcriptional misregulation in cancers	6
05203 Viral carcinogenesis	6
05160 Hepatitis C	6
05142 Chagas disease American trypanosomiasis	6
00513 Various types of N-glycan biosynthesis	5
00534 Glycosaminoglycan biosynthesis - heparan sulfate / heparin	5
04010 MAPK signaling pathway	5
04064 NF-kappa B signaling pathway	5
04020 Calcium signaling pathway	5
04810 Regulation of actin cytoskeleton	5
04650 Natural killer cell mediated cytotoxicity	5
04913 Ovarian Steroidogenesis	5
04614 Renin-angiotensin system	5
04975 Fat digestion and absorption	5
05222 Small cell lung cancer	5

04932 Non-alcoholic fatty liver disease NAFLD	5
05162 Measles	5
05145 Toxoplasmosis	5
00565 Ether lipid metabolism	4
00600 Sphingolipid metabolism	4
00230 Purine metabolism	4
00512 Mucin type O-glycan biosynthesis	4
00604 Glycosphingolipid biosynthesis - ganglio series	4
00511 Other glycan degradation	4
00983 Drug metabolism - other enzymes	4
02010 ABC transporters	4
04310 Wnt signaling pathway	4
04350 TGF-beta signaling pathway	4
04080 Neuroactive ligand-receptor interaction	4
04520 Adherens junction	4
04620 Toll-like receptor signaling pathway	4
04612 Antigen processing and presentation	4
04920 Adipocytokine signaling pathway	4
03320 PPAR signaling pathway	4
04915 Estrogen signaling pathway	4
04977 Vitamin digestion and absorption	4
04360 Axon guidance	4
05206 MicroRNAs in cancer	4
05215 Prostate cancer	4
05323 Rheumatoid arthritis	4
05010 Alzheimer's disease	4
05410 Hypertrophic cardiomyopathy HCM	4
05416 Viral myocarditis	4
05161 Hepatitis B	4
05169 Epstein-Barr virus infection	4
05146 Amoebiasis	4
00100 Steroid biosynthesis	3
00603 Glycosphingolipid biosynthesis - globo series	3
04012 ErbB signaling pathway	3
04390 Hippo signaling pathway	3
04146 Peroxisome	3
04530 Tight junction	3
04666 Fc gamma R-mediated phagocytosis	3
04910 Insulin signaling pathway	3
04918 Thyroid hormone synthesis	3
04270 Vascular smooth muscle contraction	3
05218 Melanoma	3
05020 Prion diseases	3
05412 Arrhythmogenic right ventricular cardiomyopathy ARVC	3
05414 Dilated cardiomyopathy DCM	3
05120 Epithelial cell signaling in Helicobacter pylori infection	3
05100 Bacterial invasion of epithelial cells	3
00030 Pentose phosphate pathway	2
00052 Galactose metabolism	2
00500 Starch and sucrose metabolism	2

00140 Steroid hormone biosynthesis	2
00590 Arachidonic acid metabolism	2
00240 Pyrimidine metabolism	2
00480 Glutathione metabolism	2
00514 Other types of O-glycan biosynthesis	2
00532 Glycosaminoglycan biosynthesis - chondroitin sulfate / derma	2
00533 Glycosaminoglycan biosynthesis - keratan sulfate	2
00563 Glycosylphosphatidylinositol GPI -anchor biosynthesis	2
00740 Riboflavin metabolism	2
00900 Terpenoid backbone biosynthesis	2
00627 Aminobenzoate degradation	2
03013 RNA transport	2
04370 VEGF signaling pathway	2
04210 Apoptosis	2
04672 Intestinal immune network for IgA production	2
04062 Chemokine signaling pathway	2
04916 Melanogenesis	2
04972 Pancreatic secretion	2
04978 Mineral absorption	2
04724 Glutamatergic synapse	2
04727 GABAergic synapse	2
04725 Cholinergic synapse	2
04728 Dopaminergic synapse	2
04722 Neurotrophin signaling pathway	2
05210 Colorectal cancer	2
05214 Glioma	2
05221 Acute myeloid leukemia	2
05220 Chronic myeloid leukemia	2
05211 Renal cell carcinoma	2
05219 Bladder cancer	2
05213 Endometrial cancer	2
05321 Inflammatiory bowel disease IBD	2
05014 Amyotrophic lateral sclerosis ALS	2
05132 Salmonella infection	2
05140 Leishmaniasis	2
05143 African trypanosomiasis	2
01200 Carbon metabolism	1
01212 Fatty acid metabolism	1
01230 Biosynthesis of amino acids	1
00010 Glycolysis / Gluconeogenesis	1
00040 Pentose and glucuronate interconversions	1
00051 Fructose and mannose metabolism	1
00562 Inositol phosphate metabolism	1
00680 Methane metabolism	1
00062 Fatty acid elongation	1
01040 Biosynthesis of unsaturated fatty acids	1
00330 Arginine and proline metabolism	1
00440 Phosphonate and phosphinate metabolism	1
00601 Glycosphingolipid biosynthesis - lacto and neolacto series	1
00780 Biotin metabolism	1

00790 Folate biosynthesis	1
00860 Porphyrin and chlorophyll metabolism	1
00944 Flavone and flavonol biosynthesis	1
00232 Caffeine metabolism	1
00363 Bisphenol degradation	1
00980 Metabolism of xenobiotics by cytochrome P450	1
03060 Protein export	1
02020 Two-component system	1
04330 Notch signaling pathway	1
04391 Hippo signaling pathway -fly	1
04150 mTOR signaling pathway	1
04110 Cell cycle	1
04115 p53 signaling pathway	1
04540 Gap junction	1
04621 NOD-like receptor signaling pathway	1
04660 T cell receptor signaling pathway	1
04662 B cell receptor signaling pathway	1
04664 Fc epsilon RI signaling pathway	1
04911 Insulin secretion	1
04912 GnRH signaling pathway	1
04914 Progesterone-mediated oocyte maturation	1
04917 Prolactin signaling pathway	1
04970 Salivary secretion	1
04971 Gastric acid secretion	1
04973 Carbohydrate digestion and absorption	1
04962 Vasopressin-regulated water reabsorption	1
04960 Aldosterone-regulated sodium reabsorption	1
04964 Proximal tubule bicarbonate reclamation	1
04966 Collecting duct acid secretion	1
04726 Serotonergic synapse	1
04626 Plant-pathogen interaction	1
05204 Chemical carcinogenesis	1
05212 Pancreatic cancer	1
05216 Thyroid cancer	1
05217 Basal cell carcinoma	1
05223 Non-small cell lung cancer	1
05320 Autoimmune thyroid disease	1
05330 Allograft rejection	1
05332 Graft-versus-host disease	1
05340 Primary immunodeficiency	1
05016 Huntington's disease	1
05030 Cocaine addiction	1
05031 Amphetamine addiction	1
05034 Alcoholism	1
04940 Type I diabetes mellitus	1
04930 Type II diabetes mellitus	1
05110 Vibrio cholerae infection	1
05131 Shigellosis	1
05134 Legionellosis	1
